# In Vitro Methods for Measuring the Permeability of Cell Monolayers

**DOI:** 10.3390/mps5010017

**Published:** 2022-02-09

**Authors:** Radoslaw Bednarek

**Affiliations:** Department of Cytobiology and Proteomics, Medical University of Lodz, 92-215 Lodz, Poland; radoslaw.bednarek@umed.lodz.pl; Tel.: +48-42-272-57-36

**Keywords:** permeability, endothelium, epithelium, dysfunction, transendothelial resistance, fluorescent tracer, microfluidics

## Abstract

Cell monolayers, including endothelial and epithelial cells, play crucial roles in regulating the transport of biomolecules to underlying tissues and structures via intercellular junctions. Moreover, the monolayers form a semipermeable barrier across which leukocyte transmigration is tightly regulated. The inflammatory cytokines can disrupt the epithelial and endothelial permeability, thus the reduced barrier integrity is a hallmark of epithelial and endothelial dysfunction related with numerous pathological conditions, including cancer-related inflammation. Therefore, the assessment of barrier function is critical in in vitro models of barrier-forming tissues. This review summarizes the commercially available in vitro systems used to measure the permeability of cellular monolayers. The presented techniques are separated in two large groups: macromolecular tracer flux assays, and electrical impedance measurement-based permeability assays. The presented techniques are briefly described and compared.

## 1. Introduction

The epithelial and endothelial tissues constitute the barriers of fundamental physiological significance. They provide the compartmentalization of human organisms in order to guarantee the distinct conditions in separated compartments [1]. These defensive cellular walls separate human organisms from the environment, guarding them from harmful factors, including pathogenic microorganisms. Thus, the bacterial and viral pathogens meet these barriers as the obstacles on their way to the nutrient-abundant environment on the other side of the barrier. Consequently, the certain disease states, including bacterial infections, allergies, immune-system hyper-responsiveness, inflammation, diabetes, cancer and genetic diseases, target the epithelial and endothelial barrier function. The molecular mechanisms triggered by bacterial and viral pathogens, as well as other diverse processes accompanying the diseases, make these barriers weaker, thus leading to their breakdown. Therefore, the dysfunction of these size-selective and semi-permeable filters of plasma proteins, solutes and fluids results in the uncontrolled leak of physiological fluids [2]. The barrier function is maintained by a combination of epithelial or endothelial cell monolayers, polarized into the apical (directed into the luminal spaces) and basolateral (directed into basal lamina) membrane, and four types of cell junctions: tight junctions, adherens junctions, gap junctions and desmosomes. Among them, the tight junctions (TJ) play the most considerable role in barrier tightening. Some of the TJ proteins are associated with actin filaments and form the cytoplasmic polarity complex: ZO-1, ZO-2, ZO-3, cingulin, AF-6 and 7H6 [1,3]. The transmembrane proteins of TJ, including claudins, occludin and junctional adhesion molecules (JAMs), form a zipper-like structure that tightly links the neighboring cells [3]. The research of our laboratory is particularly focused on a TJ protein that is known as the F11 receptor or the junctional adhesion molecule-A (F11R/JAM-A), which is expressed constitutively on the membrane surface of human platelets [4] and at the TJ of vascular endothelial and epithelial cells where homophilic interactions between F11R/JAM-A molecules are formed [5].

In our recent study, we utilized the F11R/JAM-A peptide antagonist P4D that disturbs the homophilic interactions between F11R/JAM-A molecules located on the surfaces of two different, adjacent cells and thus inhibits the tight junctions formation to demonstrate the role of F11R/JAM-A in breast cancer metastasis [6]. We have shown, that the F11R/JAM-A antagonistic peptide directly affects the epithelial and endothelial barrier permeability, since it blocks the homophilic interactions of F11R/JAM-A tight junction protein in a highly specific mode. This is an example that barrier permeability can also be a drug target, but the interactions of a drug with cellular barrier should be strictly controlled and carefully monitored during the trials. Consequently, there is a considerable need to estimate the dysfunction of the epithelial or endothelial barrier in a standardized quantitative mode. This review will focus on several in vitro experimental model systems those mimic the in vivo biological barriers.

The barrier dysfunction is characterized the most frequently by two major parameters, including the macromolecular permeability, which is a direct indicator of the solute flux across the cellular barrier, and electrical resistance, which reports the tightness of cellular monolayer for ion flow [2]. The macromolecular permeability is determined with a fluorescently labeled macromolecular tracer, including dextrans and polystyrene microspheres. The transendothelial or transepithelial electrical resistance (TEER) is a measure of ionic permeability through intercellular clefts. Thus, the TEER and FITC-dextran flux are two distinct measures of permeability that characterize different parameters, that can be regulated independently [7]. These two major parameters are the base to distinguish two large groups for the barrier permeability assessment techniques. This review, however, does not describe the custom experimental systems that are not commercially available, including the microfluidic device-based assays [8].

## 2. Overview of Permeability Assays

### 2.1. Macromolecular Tracer Assays

**Figure 1:**
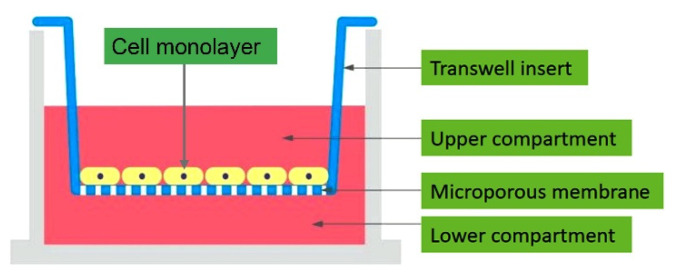
The overview of a transwell support system (taken from https://tumomics.creative-proteomics.com/, accessed on 28 June 2021, with permission).

Macromolecular permeability of cellular monolayers is the most frequently analyzed by a group of tracer flux assays, due to their comparably low costs and technical simplicity. These techniques can be collectively termed as macromolecular tracer assays, tracer flux assays, or permeation/diffusion assays [9]. This experimental approach is used to measure the transepithelial or transendothelial transport in the absence of hydrostatic or osmotic pressure gradients. The assay utilizes the cell culture inserts, termed as the transwell support system, in which the epithelial or endothelial cells are cultured to form a confluent monolayer on a microporous semipermeable membrane filter (Figure 1). The role of the membrane is to support the cell layer mechanically, without acting as a significant diffusion barrier. The membrane is then placed between two fluid compartments so that any flux of solutes from one compartment to the other is required to pass through the interfacial cell layer. In the other words, the two compartments communicate by the cell-covered membrane. The upper compartment represents the apical (luminal) side, while the lower compartment represents the basolateral (abluminal) side of the epithelium (endothelium) [10]. The transwell support systems include the polycarbonate (PC), polyester (PE) or polyethylene terephthalate (PET) micropore (usually 0.4 μm or 3 μm diameter) membranes in transwell inserts placed in a multiwell (6, 12 or 24) plates. The cells are seeded at a specified number per insert depending on the cell line, incubated in a humidified incubator with the temperature of 37 °C and 5% CO_2_ atmosphere to reach confluency, and subjected to a suitable treatment, while the non-treated cells serve as a control sample. The cells can be seeded directly on PC, PE or PET membranes or on such membranes coated with collagen type I, collagen type IV, gelatin, fibronectin or a mixture of collagen and fibronectin [11]. At the specified time after the cell treatment, the particles of a labeled macromolecular tracer are added into the upper compartment (donor), and the culture medium from the lower compartment (acceptor) is replaced with PBS or another transparent buffer. The samples are then collected from the acceptor compartment after the defined time intervals, depending on a tracer compound. The increase in the tracer concentration in the acceptor compartment is directly proportional to the barrier permeability of the cell monolayer.

The macromolecular tracers include urea, mannitol, proteins (e.g., albumin), polysaccharides (e.g., dextran and inulin) or polyethylene glycol (PEG) of a defined molecular weight, or microspheres of a defined diameter. The most frequently chosen tracer molecule for the assay is dextran, a glucose polymer, because it is well established as a marker and represents a suitable molecule for fluorescent labeling due to its easily accessible hydroxyl and carbonyl groups [12]. Moreover, the desirable tracers for the essay should be highly water-soluble, non-membrane permeable and not actively transported by the cells. All of these requirements are fulfilled by the dextrans with a molecular weight range mostly from 3 up to 70 kDa [8,10]. To obtain the reliable experimental results, the tracer of a defined molecular weight should be used, for example, 40 kDa dextran, since the molecular weight of the substance transported through the epithelial or endothelial barrier considerably affects the permeability rate [13].

However, the cellular permeability can be characterized with the greater accuracy when a mixture of oligomers with different size is applied. The mixture should contain the oligomers with a precisely defined range of molecular weights. The sophisticated technique, with the use of a continuous series of PEG oligomers (with a range from dimer to 30-mer), which allows for the characterization of the paracellular permeability at sub-Ångstrom increments, is called PEG profiling and was described by Van Itallie and Anderson [14]. Similarly, the APTS-Dextran Ladder is the laboratory tool that uses the mixture of dextrans with different molecular weights (containing from 1 to 35 glucose units) labeled by reductive amination with fluorescent 8-aminopyrene-1,3,6-trisulfonate (APTS) [12]. The resolution of the APTS-Dextran Ladder is comparable to that provided by PEG profiling.

The tracer molecules should be labeled to enable the measurement of the tracer concentration in the acceptor compartment. The first published description of the technique reported the use of sucrose with a molecular weight of 342 Da labeled with carbon-14 for the measurement of the flux on a monolayer of brain endothelial cells cultured on a collagen-coated nylon mesh [15]. Later on, the radioactively labeled protein tracers were used, e.g., ^125^I-labeled bovine serum albumin, as reported in the paper published in 1987 by Cooper et al. that included one of the first thorough descriptions of this experimental technique [16]. Another study was performed with the use of radioisotope-labeled polysaccharide, ^3^H-inulin [17]. Currently, the macromolecular tracers are labeled predominantly with fluorophores and include dextran labeled with fluorescein isothiocyanate (FITC-dextran), dextran labeled by reductive amination with 8-aminopyrene-1,3,6-trisulfonate (APTS-dextran) [12], dextran labeled with rhodamine [18] and FITC-labeled polystyrene microsphere (FluoSpheres^®^, 10–15 μm diameter) beads [19].

Another way to study the monolayer permeability is the use of fluorophores as the stand-alone low molecular fluorescent tracers [8]. The commonly used compounds include fluorescein, which can be used for the monocarboxylic acid transporter-mediated pathway, and rhodamine 123, which can be used as a model molecule for the p-glycoprotein-mediated efflux pathway [20]. A fluorophore used as a small molecule paracellular transport marker is Lucifer Yellow with a molecular mass of 0.44 kDa. The transcellular passage of Lucifer Yellow may also occur via organic anion transporters [8].

After incubation in darkness (about 1 h), the buffer from the wells outside the inserts was transferred into the wells of a black 96-well plate and the fluorescence was measured using a microplate reader at 485–492 nm of excitation and 517–520 nm of emission wavelengths, when FITC was used as a label. The intensity of fluorescence was directly proportional to the barrier permeability. Macromolecule diffusion across the epithelial or endothelial monolayers can also be quantified using enzymatic markers, including horseradish peroxidase [21]. Moreover, the macromolecular diffusion can be analyzed by detailed visualization using confocal microscopy [22] or electron microscopy [23]. The increase in the tracer concentration in the acceptor compartment enables the calculation of the permeability coefficient *P_E_*, which characterizes the barrier properties of the studied cell monolayer [9]:

(1) $$J=P_{E}\times \Delta c=P_{E}\times \left(c_{Donor}-c_{Acceptor}\right),$$

where *J* is the area solute flux across the monolayer expressed in [mol/(cm^2^ × s)], while Δ*c* is the initial concentration gradient between both compartments expressed in [mol/cm^3^]. Consequently, the unit of the permeability coefficient *P_E_* is [cm/s]. Thus, *P_E_* represents the permeability of the filter covered with the cell monolayer and has to be corrected for the permeability of the cell-free filter membrane *P_F_*. For such a correction, the experimentally obtained permeability coefficient *P_EXP_* is corrected by the permeability of the cell-free filter *P_F_* according to the following equation to estimate the true *P_E_* value of the cell monolayer [9]:

(2) $$\frac{1}{P_{E}}=\frac{1}{P_{EXP\;}}-\frac{1}{P_{F}},$$

The archetype of the transwell system was initially developed for growing confluent primary cultured monolayers of guinea pig gastric mucous cells and described for the first time in 1985, but the insert contained a central hole with a collagen gel instead of a micropore membrane filter [24]. This system was designed at that time to fit in Ussing chambers and to be suitable for electrophysiological, ion transport and pharmacological studies. Nevertheless, the possible use of the system as an important tool in barrier function research was also suggested.

The precursory paper by Cooper et al. contains the detailed morphological analysis of endothelial monolayer grown on the surface of membrane filter [16]. The analysis was performed by light microscopy and transmission electron microscopy. The en face phase microscopy study shows the monolayer confluence over the entire surface of the membrane filter. The cross-sectional light and electron microscopic appearance of the monolayer was performed after the control treatment (culture medium) and oleic acid treatment. In the control monolayer, the cells were confluent over the surface and were nonvacuolated. Moreover, the areas of junctional specialization could be noticed. The cells subjected to oleic acid treatment were round and vacuolated, but the formation of gaps at intercellular junctions was not observed. These observations were confirmed later by another light and electron microscopy study of the human umbilical vein endothelial cell (HUVEC) monolayers [17]. Phase contrast microscopy of the cell monolayer grown on transwell devices is impossible due to the inability of light to pass through micropore membranes, thus the cell monolayer was stained by the Diff-Quik procedure for light microscopy visualization. The electron microscopic analysis of the HUVEC cell monolayers revealed that, in addition to being confluent, the cells retain morphological characteristic similar to those when the cells are grown on tissue culture polystyrene. The HUVECs were observed to plate up to the edge of the membrane, and no intercellular spaces were present through which the underlying membrane could be seen [17].

While preparing the conditions for the tracer flux assay, several critical points must be taken into account [25]. These factors include the selection of the proper cell line, the cell culture medium that provides the desired conditions of the assay (solute concentration, pH, temperature, presence or absence of a metabolic source of energy or ions, presence or absence of proteins that can potentially bind the solute, presence or absence of competing solutes), and the application of the solute to the apical or basolateral side of the monolayer. Moreover, the correct microporous membrane for the monolayer cell culture should be carefully selected in terms of pore size and surface area. The nature and thickness of the optionally applied supporting matrix layer, e.g., collagen, should also be considered. The ideal diffusion characteristics of macromolecular permeability assay system occur when the major diffusion barrier is provided by the cell monolayer and not the microporous membrane or the supporting matrix. The control samples without the cell monolayer should be included into an experimental design (the microporous membrane alone and the microporous membrane coated with the supporting matrix to ensure that the diffusion barrier is provided solely by the cell monolayer, while the solute freely permeates through the membrane and the matrix). The correct regulation and optimization of all the mentioned factors should provide the best possible imitation of the barrier in vivo.

### 2.2. Resistance-Based Assays

#### 2.2.1. Transepithelial/Endothelial Electrical Resistance (TEER) Measurements

The use of macromolecular tracer compounds can interfere with the studied transport process and can alter the barrier integrity. Moreover, following the treatment with the chemicals used for a tracer assay, the cells cannot be used for further experiments. Thus, the noninvasive techniques were applied instead for the continuous monitoring of the barrier permeability [26].

The permeability of epithelial and endothelial cell monolayers is commonly analyzed by the noninvasive measurements of transepithelial/endothelial electrical resistance (TEER). In electrophysiology, this electrical resistance of cellular barrier is represented by R^t^, the reciprocal of the sum of the permeabilities of all ions of the adjacent bath solution times their respective concentrations [27]. The electrical resistance of a cellular monolayer, measured in ohms, is a quantitative measure of the barrier integrity. TEER expresses the resistance to an electrical current passed across the cell monolayer as a measure of permeability to small inorganic ions [13]. The classical setup for the measurement of TEER is similar to that described for the macromolecular tracer assay. Likewise, it consists of a cellular monolayer cultured on a semipermeable filter insert that defines two compartments: apical/luminal (upper) and basolateral/abluminal (lower). Two electrodes separated by the cellular monolayer were used for resistance measurements: the first one was placed in the upper compartment and the other in the lower compartment. The resistance was calculated using Ohm’s law:

(3) $$R=\frac{U}{I}$$

where *R* is the resistance (measured in ohms, Ω), *U* is the voltage (measured in volts, V) and *I* is the current (measured in amperes, A). The evaluation procedure includes the blank resistance measurement (*R_BLANK_*) of the microporous membrane only (without cells) and the measurement of the resistance across the cell monolayer on the membrane filter *R_TOTAL_*, that includes the cell layer resistance *R_TEER_*, the resistance of the culture medium *R_M_*, the membrane insert resistance *R_I_*, and the resistance of the electrode medium interface *R_EMI_*. The cell-specific resistance *R_TISSUE_* can be obtained as:

(4) $$R_{TISSUE}=R_{TOTAL}-R_{BLANK}$$

*TEER* values are reported in units of Ω × cm^2^ (*TEER_REPORTED_*) and calculated as:

(5) $$TEER_{REPORTED}=R_{TISSUE}\times M_{AREA}$$

where *M_AREA_* is the area of the semipermeable membrane (measured in cm^2^). The *TEER* value is inversely proportional to the permeability of the epithelial and endothelial cell monolayer.

*TEER* quantifications can be performed with the use of several commercially available systems, which are described below. These systems guarantee the noninvasive measurements and can evaluate the barrier integrity of epithelial or endothelial cells at various stages of differentiation and growth [26].

##### Epithelial Volt/Ohm Meter (EVOM)

**Figure 2:**
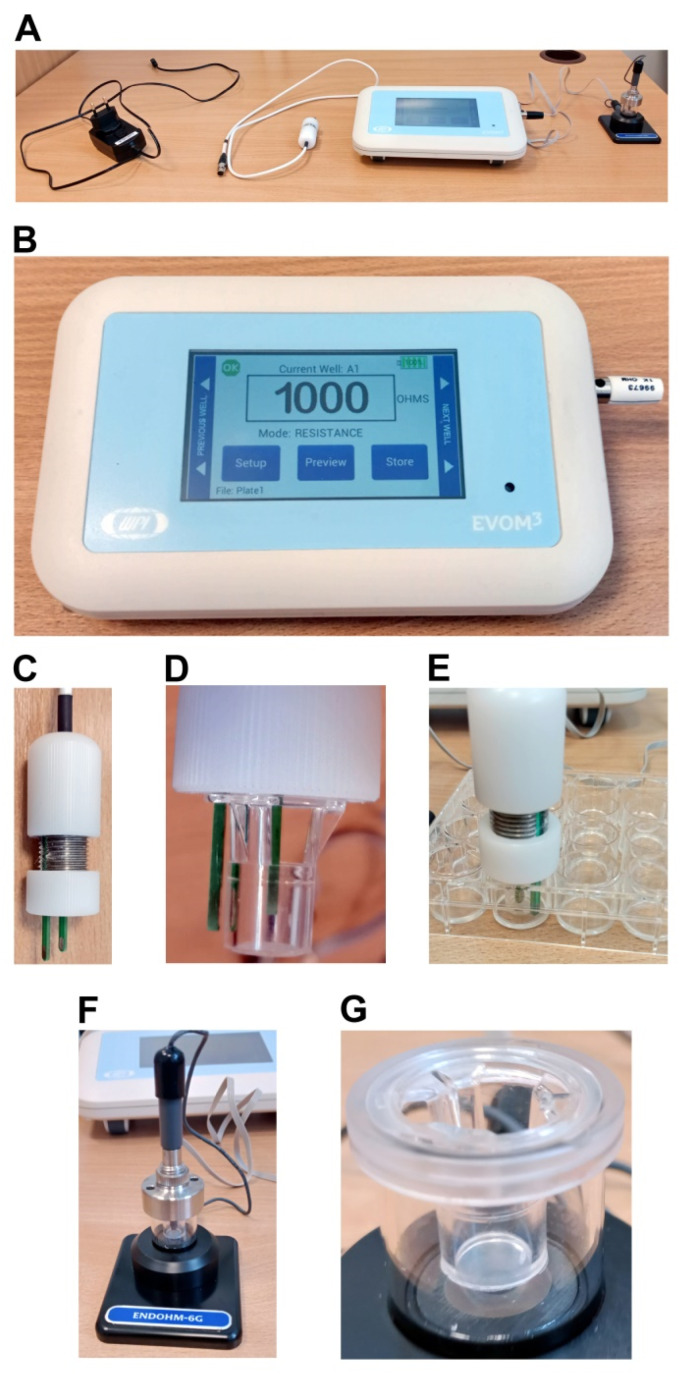
The overview of EVOM3 (epithelial volt–ohm meter) system with all its components, used for the transepithelial/transendothelial electrical resistance (TEER) measurements (from left to right: A/C power supply and charger, STX2-Plus electrode, EVOM3 meter and the optional Endohm-6G chamber electrode). (**A**) the general view of the whole EVOM3 system. (**B**) The EVOM3 meter with the 1 kOhm test resistor inserted into the electrode port: the display shows the actual resistance of the test resistor. (**C**) The body of the STX2-Plus chopstick electrode. (**D**,**E**) The proper adjustment of STX2-Plus electrode to fit the insert with a 6.5 mm diameter (compatible with 24-well plates): the outer electrode reaches close to the bottom of the well plate, while the inner electrode is close to the membrane inside the insert, without touching it. (**F**) The Endohm-6G chamber electrode with the 6.5 mm cell culture insert inside the chamber, connected with the EVOM3 meter by the Endohm cable. (**G**) The detailed view of the Endohm-6G chamber with the cell culture insert.

The most widely used and commonly distributed TEER measurement instrument is called the epithelial volt–ohm meter (EVOM; World Precision Instruments, Sarasota, FL, USA). The newest version of the instrument is called EVOM3. The EVOM3 system with all the associated components is presented in Figure 2. Figure 2A presents the general view of the whole EVOM3 system. The EVOM3 instrument with the 1 kOhm test resistor inserted into the electrode port is shown in Figure 2B. As compared with the older versions, the EVOM3 meter was re-engineered and contains the following new features: a more stable and accurate processor, the possibility to write the data on a USB stick, the footswitch, the touch screen interface, the auto ranging resistance feature and the overrange display feature to eliminate the false readings. The EVOM3 has adjustable current levels in three fixed ranges with two lower ranges for the sensitive membranes and high resistance ranges of up to 100 KΩ. The EVOM3 instrument is equipped with a four electrode sensor. Two electrodes are responsible for current sourcing and the other two for the voltage measurement. The EVOM3 instrument passes a known constant current through the membrane on two electrodes. Then, the voltage needed to pass that current on the other two electrodes is measured and the resistance is computed by the instrument in accordance with Ohm’s law. The changes of the current polarity from a positive current to negative current are performed 12.5 times per second, which corresponds with the alternating current (AC) frequency of 12.5 Hz. These polarity changes prevent a charge formation on the membrane and negate any voltage offsets due to the membrane potential or from the electrodes. The amount of AC current is low: 2, 4 or 10 µA. This minimizes any unwanted or accidental activation of the epithelial or endothelial cell monolayer and prevents the migration of metal ions.

Before the measurement procedure, the EVOM3 device has to be calibrated with the use of the 1 kOhm resistor—the display has to show the reading of 1000 Ohms (Figure 2B). Otherwise, the proper reading has to be set by the “calibration” procedure due to the manufacturer’s instructions. The measurements are performed with the use of a chopstick electrode or a measurement chamber. The EVOM3 system uses the STX2-Plus electrode, which is compatible with 12-well (diameter: 12 mm) and 24-well (diameter: 6.5 mm) transwell inserts. Thus, the cell monolayer has to be cultured and treated on the inserts. The electrode has to be placed correctly for accurate and reliable measurements. This is facilitated by the unequal lengths of the electrodes (Figure 2C–E). The longer, external electrode touches the bottom of the insert containing the external culture media. The shorter, internal electrode is then prevented from reaching the bottom of the insert. Thus the proper repeatable positioning is provided, which improves the reproducibility of the measurements. For the 24-well plates, the adjustment ring can be set to keep the electrode on the outer edge of a well and the electrode can sit securely on the insert that is placed inside the well (Figure 2E). However, for 12-well plates, the electrode has to be hand held, which causes the significant discomfort of the measurements and can lead to limited reproducibility.

In practice, the correct positioning of the STX2-plus electrode during the measurements is difficult. This problem is omitted when the measurements are performed with the use of the Endohm electrode chamber (Figure 2F). This device contains a pair of concentric electrodes that incorporate a voltage-sensing Ag/AgCl pellet in the center and the silver annular current electrode coated with silver chloride (gray). The top cap assembly includes the cap, locking nut and electrode. The cap centers the electrode in the culture cup, while the locking nut fixes the height of the electrode in the chamber. For the measurement, the cell culture inserts are transferred from their culture wells and placed inside the Endohm chamber, which eliminates the laborious positioning, as in the case of a chopstick electrode (Figure 2G). This ensures the reproducibility of the measurements. The accuracy of the Endohm chamber is greater than that of the STX2-Plus electrode, since the concentric pairs of the electrodes applied above and beneath the insert membrane results in the reduction in the background resistance from 150 Ω (as for STX2-Plus electrode) to <2 Ω. The shape of the current electrodes allows for a more uniform current density to flow across the membrane. The fixed centered electrode geometry of the Endohm chamber results in the 1–2 Ω variation of readings on the same sample, as compared to 5% of the total reading using the STX2-Plus electrodes. Consequently, the Endohm chambers are recommended for a more precise measurement, for example, for the low-resistance tissue cell culture, especially the endothelial cell monolayers. The Endohm chambers are available in different diameters: Endohm-6 for the 6.5 mm culture cap (24-well), Endohm-12 for the 12 mm culture cup (12-well) and Endohm-24SNAP for the 24 mm culture cap (6-well) and COSTAR Snapwell^TM^ culture cup.

#### 2.2.2. Real-Time Cell Electrical Impedance Sensing

The changes in the TEER of a cellular barrier can be monitored in real-time. The devices that enable real-time measurements are powered with the alternating current from the mains, thus they in fact measure the barrier’s impedance, which is defined as the resistance to alternating currents (ACs). It is a complex physical quantity that is dependent on the AC frequency, since the latter determines the current pathway across the cell layer: transcellular vs. paracellular [28]. The macromolecular permeability of cellular monolayers can be analyzed by a group of real-time electrical impedance measuring instruments.

##### Ussing Chamber

**Figure 3:**
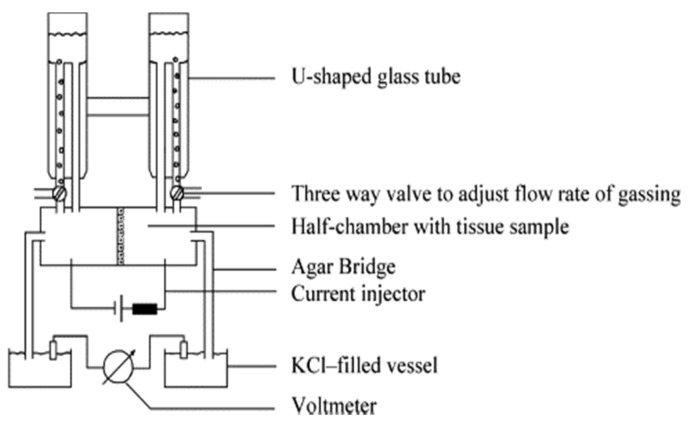
Schematic drawing of a circulating Ussing chamber (taken from [30], license no. 5112401256947).

The Ussing chamber system was originally designed by the Danish zoologist Hans Ussing in the 1950s, to study the net transport of ions through the skin of a frog [29]. It is a simple but powerful technique used to investigate ion transportation across any relevant tissue, including the endothelium and epithelium [30]. The Ussing chamber consists of the chamber itself and the electrical circuitry, which allows for the measurement of the current resistance, intensity, voltage, impedance and capacitance. There are two types of Ussing chambers: the circulating chamber and the continuously perfused chamber. The continuously perfused chamber will not be described in this review, since this is not commercially available. The circulating chamber consists of a U-shaped tubing system usually made of glass that is filled with the experimental solution (Figure 3). The tubing can be heated and is gassed with air, carbon dioxide, O_2_, or N_2_. The gassing oxygenates the solution contents and stirs the liquid to provide the complete convection, the so-called ‘bubble lift’. The U-shaped tube prevents the damage caused by the bending of the tissue, since it balances the hydrostatic pressure on both sides of the chamber. During the experimental procedure, the substances are added to one or both sides of the tube in a sequential manner, thus they are present in the solution until the end of the experiment.

Chambers are made of Teflon or Lucite and are available in different sizes and shapes. The most common application of the Ussing chamber is for the cell cultures grown on permeable supports, including the transwell inserts, those of which very often are available in the shape that matches the Ussing chamber half-cells.

The procedure of an experiment that utilizes an Ussing chamber starts with the preparation of the chambers and solutions. Then, the system without any cell/tissue culture (endothelial/epithelial monolayer) is flushed with bath solution. The temperature is adjusted to the desired value when there is no liquid leakage from the system. Next, the current and voltage electrodes are inserted into the half-cells. Depending on the electrode type (a glass column filled with potassium chloride, agar-bridge, calomel electrode) the electrode resistance can be non-constant, which can result in asymmetries. Therefore, the system is tested for noise and offset voltages as soon as the electrodes are connected to the current/voltage pulse injectors and the volt-/ammeter, respectively. The resistance of the empty chambers can be estimated by turning on the current/voltage pulses. This is necessary for the calculation of resistance and currents. Some devices enable to cancel out the resistance of the solution and the offset voltage generated by non-equilibrated electrodes. This should be performed before the cell/tissue culture or an empty (blank sample) filter is inserted. Then, the chambers are disconnected from the solution supply and the cell/tissue can be mounted. The recording begins after the system is reassembled. Immediately after the cell/tissue culture insertion, the values of all electrical parameters (voltage, intensity, resistance) are oscillating. This can result from the mechanical stress on the sample or from the accidental stimulation. Therefore, the tissue should be allowed to recover for between 10 and 40 min before the experimental treatment. When a stable baseline is obtained, the data acquisition system is switched to a higher time resolution, the initial procedures are completed and the actual experiment can be started.

The Ussing chamber technique has both its strengths and limitations [29]. One of the major limitations of the classic Ussing chamber is that the simultaneous preparation and analyses of a large set of tissue samples is impossible, thus this is a rather low throughput technique. The tissue segments in a classic Ussing chamer are incorporated in a vertical position. More recent systems, including InTESTine™ (TNO, Amsterdam, The Netherlands) or NaviCyte™ Horizontal Ussing System (Warner Instruments, Hollistion, MA, USA), allow for a horizontal placement and provide a higher efficiency. For example, the InTESTine™ device is compatible with the 6- or 24-well plate format [31], whereas the NaviCyte™ Horizontal Ussing System allows for the running pf up to 6 chambers simultaneously [32].

This experimental technique was applied in a wide array of research areas, including the study on the mechanisms of diarrheal disease in the setting of Salmonella infection and on the regulation of epithelial chloride secretion by the epidermal growth factor receptor [33], and the research on the cytotoxic effect of smoke from polymer combustion on human lungs [32]. For a long time, the Using chamber systems have been the most frequently used for the study of intestinal epithelium [34], but more recent papers describe the research on the airway epithelia, including the targeting of prostaglandin receptors in cystic fibrosis (CF) treatment [35].

##### ACEA xCELLigence^®^ Real-Time Cell Analysis (RTCA)

The real-time impedance measurements during the cell monolayer development can be performed with the ACEA xCELLigence^®^ Real-Time Cell Analysis (RTCA) system (Roche, Mannheim, Germany). The system consists of three parts: an RTCA instrument, a personal computer-based RTCA control unit and a single-use electronic multiwall plate. The RTCA instrument is available in three versions: RTCA-SP (Single Plate), RTCA-DP (Double Plate), or RTCA-MP (Multiple Plate). The following single-use multiwall plates, termed as E-Plates, are compatible with the RTCA system (16-well ‘E-Plate VIEW 16 PET’ plates or 96-well ‘E-Plate VIEW 96 PET’ plates).

The typical experimental procedure is performed as follows. The gold-film electrodes deposited on the bottom of the E-plate electrode arrays are equilibrated with a culture medium overnight. Subsequently, the medium is aspirated and replaced by a volume of fresh medium for impedance background measurements. The cells are seeded at a specified density in a suitable cell culture medium with supplements. Usually, the 30 min time is required to allow the cell sedimentation at room temperature. Afterwards, the plates are locked into the RTCA instrument for continuous recording of the impedance changes at three different alternating current (AC) frequencies (10 kHz, 25 kHz, 50 kHz), which are expressed as the cell index (*CI*) values. *CI* is a dimensionless parameter based on relative impedance changes referenced to the values of the cell-free electrode at each frequency:

(6) $$CI=\underset{i=1,\ldots ,N}{\mathrm{Max}}\left(\frac{R_{cell}\left(fi\right)}{R_{b}\left(fi\right)}-1\right),$$

where *N* is the number of the frequency points at which the impedance is measured, whereas *R_cell_*(*fi*) and *R_b_*(*fi*) are the frequency-dependent electrode resistances with cells present and without cells, respectively [36]. Thus, *CI* is a quantitative measure of cell status in a well, including the cell number, cell viability, adhesion degree and morphology. Although *CI* is measured at three frequencies, only the most sensitive readings are returned by the software as a function of time and reported as the final results. Since the frequencies are close together, the differences between the *CI*s at different frequencies are small but different. In general, *CI* values rise with the increasing coverage of the electrode with cells, which is caused at an early stage by cell sedimentation and, later on, by cell proliferation. In practice, the ‘normalized cell index’ at a certain time point is used for most experimental settings. Its values are acquired by dividing the *CI* value by the value at a reference time point.

The documented research applications of RTCA include the toxicological studies, specifically the research on myocardial toxicity [37], and the screening of the toxicity of engineered nanoparticle bio-conjugates [38]. Furthermore, the RTCA application was reported in the hepatological field [39], as well as in traditional Chinese herbal medicine [40].

##### ECIS

Another example of the laboratory equipment enabling the monitoring of cell monolayer impedance in real-time is the electric cell-substrate impedance sensing (ECIS) system [2]. The commercially available ECIS^®^-1600 R system is manufactured by Applied Biophysics (Troy, NY, USA) and consists of an ECIS^®^ instrument that measures complex impedance at variable frequencies, a 16-well array holder that is stored inside the cell culture incubator, and a computer for instrument control and data storage. The growth surface of disposable 8-well electrode arrays can be coated with an adhesive protein. Subsequently, the epithelial/endothelial cells are seeded on the electrodes. Then, the 8-well arrays are placed inside the ECIS^®^ device for impedance monitoring. The measurements can be performed at a desired AC frequency within a range of 10 Hz–100 kHz. Typically, the exposure of cell monolayers to a substance of interest is performed after reaching constant impedance values (usually 12 to 24 h after seeding), which indicates the cell layer confluence. The impedance values of each well are then recorded at the desired time intervals during the entire analysis. The impedance values are usually presented as recorded along the time course of the experiment normalized to the impedance values immediately before the addition of modulators or test compounds. The main advantage of the ECIS system over the RTCA xCELLigence technique is that the ECIS measurements can deconvolute data into biological parameters, while the xCELLigence readouts measure only the relative total impedance values [39].

ECIS enables the study of cell proliferation, migration, differentiation, toxicity and monolayer barrier integrity. Similar to the RTCA technique, the ECIS system is used for hepatological research [39]. Moreover, this technique was applied for preclinical kidney studies [41], as well as in a wide array of other fields, including cancer research, drug screening, pathology, food safety detection, the transendothelial migration of leukocytes and cancer cells, the migration of pathogens and monitoring the activity of neuronal cells [42,43].

## 3. Conclusions

The two major parameters of the epithelial/endothelial barrier dysfunction are quantitated by the two distinct methodological groups. The macromolecular permeability characterizes the flux of high molecular weight biomolecules across the cellular barrier, including proteins, peptides, lipids, polysaccharides and nucleic acids. This parameter can be determined with the use of fluorescently labeled macromolecular tracers, with the 3-kDa or 40-kDa dextran as the most common example. The transendothelial/transepithelial electrical resistance (TEER) is a measure of the tightness of the cellular monolayer for the flow of small inorganic molecules, mostly ions.

These two methodological groups for the assessment of the cell monolayer permeability are related with two different cellular parameters, which are regulated independently. Therefore, a researcher cannot simply replace a macromolecular permeability assay with a TEER measurement. Instead, these two techniques should be used simultaneously to obtain the complex characterization of the observed effect of an experimental setup on the epithelial/endothelial barrier. On the other hand, the resistance/impedance measurements that characterize the ion flux across the cellular barrier can be performed as the end point measurements using the EVOM system. Moreover, the impedance fluctuations can be observed in real-time by the implementation of the Ussing chamber, or xCELLigence and ECIS systems, which provides the higher scientific significance than the end-point measurements by the EVOM instrument. However, in the case of the Ussing chamber, only one set of experimental conditions can be tested for each experiment, whereas the last two provide the simultaneous targeting of multiple analytes. On the other hand, it is a challenge to purchase the xCELLigence and ECIS systems due to their high prices. The Ussing chamber is a much more complicated system, both in terms of the experimental manipulations and measurement procedures, than EVOM. Thus, for simple, basic analyses, and when the high costs make the purchase impossible, the EVOM system can be the methodological approach of choice for the ion flux characterization. Otherwise, the xCELLigence and ECIS systems are highly recommended, whereas the Ussing chamber system has an additional possibility to be applied not only for in vitro experiments, but also for ex vivo and in vivo studies.
